# Management of acute uncomplicated diverticulitis without antibiotics: compliance and outcomes -a retrospective cohort study

**DOI:** 10.1186/s12873-022-00584-x

**Published:** 2022-02-21

**Authors:** Najia Azhar, Hager Aref, Adam Brorsson, Marie-Louise Lydrup, Fredrik Jörgren, Johannes Kurt Schultz, Pamela Buchwald

**Affiliations:** 1 Department of Clinical Sciences Malmö, Lund University, Malmö, Sweden; 2 Department of Surgery, Skåne University Hospital, Malmö, Sweden; 3 Department of Surgery, Helsingborg Hospital, Helsingborg, Sweden; 4 Department of Digestive Surgery, Akershus University Hospital, Lørenskog, Norway

**Keywords:** Diverticulitis, Compliance, Antibiotics

## Abstract

**Methods:**

Recent randomized control trials (RCTs) have confirmed that antibiotics in acute uncomplicated diverticulitis (AUD) neither accelerate recovery nor prevent complications or recurrences.

A retrospective cohort study was conducted, including all consecutive AUD patients hospitalized 2015- 2018 at Helsingborg Hospital (HH) and Skåne University Hospital (SUS), Sweden. HH had implemented a non-antibiotic treatment protocol in 2014 while SUS had not. Main outcomes were proportion of patients treated with antibiotics, complications, recurrences, and adherence to routinely colon evaluation.

**Results:**

A total of 583 AUD patients were enrolled, 388 at SUS and 195 at HH. The diagnosis was CT-verified in 320 (83%) vs. 186 (95%) patients respectively (*p* < 0.001). Forty-three (11%) and 94 (48%) of patients respectively did not receive antibiotics during hospitalization (*p* < 0.001). CRP was higher in the antibiotic group compared to the non-antibiotic group, both at admission and peak (90 mg/L vs 65 mg/L; *p* = 0.016) and (138 mg/L and 97 mg/L; *p* < 0.001). There were no significant differences in recurrences (22.0% vs. 22.6%; *p* = 0.87) and complications (2.5% vs. 2.9%; *p* = 0.77) between the antibiotic/non-antibiotic groups.

**Conclusion:**

The structured treatment protocol led to reduced antibiotic use and a higher standard of care in terms of CT-verification. Clinicians’ compliance to the treatment protocol and best clinical practice was poor and warrants further studies.

## Introduction

Acute uncomplicated diverticulitis (AUD) is common and has a rising incidence in the Western world, especially in younger age-groups [1–3]. In addition, AUD imposes a significant burden on health-care budgets and resource utilization [4–6]. The standard treatment of AUD has long been antibiotics, analgesics, intravenous fluids and bowel rest. Recent randomized control trials (RCTs) have confirmed that antibiotics do neither accelerate recovery nor prevent complications or recurrences in AUD [5–7]. A non-antibiotic approach is found to be cost-effective and safe in the short and long term [8]. Recent guidelines [9, 10], supported by meta-analyses [11, 12], recommend avoiding antibiotics in otherwise healthy AUD patients and reserve their use for complicated diverticulitis. Yet, many centres are still using antibiotics routinely [13, 14]. Reasons for this may be tradition but also the unclear etiology of diverticulitis, where previous theories have thought the cause being bacterial, despite little evidence supporting this [15].

In 2014, a new treatment protocol (omitting antibiotics) was introduced at Helsingborg Hospital (HH) Sweden [16], where the exclusion criteria were similar to those in the AVOD-study (Antibiotika Vid Okomplicerad Divertikulit) [6]. Complications were rare, and compliance to the protocol was 60%. However, Skåne University Hospital (SUS), the main teaching hospital in the region, had not established a similar protocol at that time, but relied on best clinical practice.

Implementation of treatment protocols or guidelines have been sparsely studied previously. A successful introduction of guidelines involves three steps: development, dissemination and implementation of guidelines [17]. There are several barriers to implementation, and they can be divided into three main factors namely personal, (related to physicians’ knowledge and attitudes), guideline-related, and external factors [18].

This study aimed to compare the in-patient management of AUD patients and treatment outcomes at two different hospitals in Sweden. We hypothesized that the treatment protocol at HH facilitated the implementation of non-antibiotic management of AUD.

## Materials and methods

The present retrospective cohort study included all consecutive in-patients >18 years with the main diagnosis of AUD admitted to HH and SUS, from January 1, 2015 to December 31, 2017.

Primary outcome was proportions of patients treated without antibiotics. Secondary outcomes were complications, recurrences, length of hospital stay and colon examination after discharge.

Patients were identified from the hospitals’ administrative system by the discharge ICD-10 code K57.3. The patient files and CT reports of all identified individuals were reviewed, and patients were only included if the diagnosis of AUD either was verified by CT or if the clinical examination, patient history and blood tests showing an inflammatory response supported the diagnosis. Criteria for AUD diagnosis on CT were acute inflammation confined to the colonic wall and/or the surrounding fatty tissue in the absence of complications such as, abscess, fistula, stricture, bowel obstruction, or peritonitis with perforation. Only the first diverticulitis admission of each patient during the period was included. Additional admissions were recorded as recurrences. Exclusion of patients were made based on the following criteria: patients with acute complicated diverticulitis at admission, patients presenting with general peritonitis or sepsis, immunosuppressed patients (defined as corticosteroids intake, ongoing chemotherapy, or having a prior transplant) and patients with ongoing antibiotic treatment at the time of admission.

HH is a teaching hospital with a catchment area covering 350 000 inhabitants. The catchment area of SUS has approximately 750 000 inhabitants. SUS consists of two separate hospitals in two different cities (Malmö and Lund). At all hospitals, the patients were first seen by emergency doctors or surgical residents at the emergency department, before being admitted to the Department of Surgery where they were managed by surgeons or gastroenterologists.

Medical charts were reviewed using the Melior patient database, and data on predetermined variables were collected. Study variables included age at admission, gender, intercurrent diseases, Charlson comorbidity index [19], previous diverticulitis before admission, body mass index (BMI), admission and peak values of P-CRP, WBC and temperature, length of hospital stay, CT-confirmation of diagnosis, if antibiotics were administrated at any time during hospitalization and whether a follow-up colon evaluation was performed within six months of the AUD episode. Antibiotics given at any time during hospitalization, rendered inclusion in the antibiotics group. Antibiotics used were commonly i.v. Cefotaxim and Metronidazole, or Piperacillin/Tazobactam for 1-3 days, followed-up by oral Ciprofloxacin or Bioclavid with Metronidazole for a total of 10 days.

All patients were followed by medical chart review for a minimum of one year after discharge in terms of recurrence of diverticulitis (defined as new acute diverticulitis episode >30 days after admission), development of complications (abscess, fistula, perforation or stenosis) and AUD related operations. Antibiotic treatment, treatment outcomes and compliance to colon evaluation were compared between HH and SUS. A multivariable regression analysis was performed, using the variable “hospital” as a proxy for protocol use. STrengthening the Reporting of OBservational studies in Epidemiology (STROBE) guidance for reporting of observational studies was followed.

All statistical analyses were performed using SPSS (Version 25, IBM, Armonk, NY, USA). Categorical variables were analysed using Pearson’s Chi2-test. Independent sample T-test was used for continuous variables that was normally distributed and Mann Whitney-U-test for variables not normally distributed. A *P*-value of < 0.05 was considered significant.

Ethical approval was obtained from a local ethical committee (Dnr 2018/980).

## Results

**Fig. 1:**
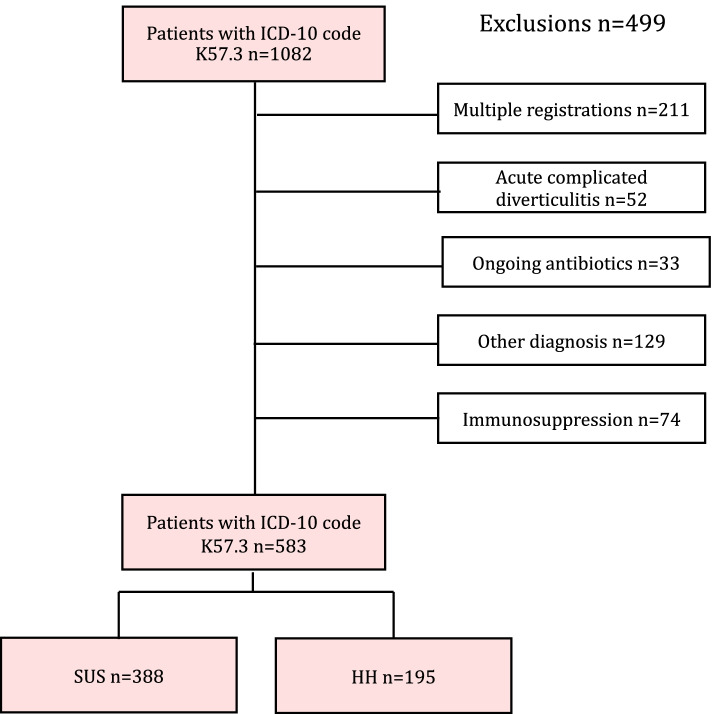
Flow chart describing inclusion and exclusion of patients with acute uncomplicated diverticulitis hospitalized at SUS and HH between 2015-2017

A total of 1082 admissions registered with the ICD-10 code K57.3 were identified. After excluding duplicates and applying the exclusion criteria, the final cohort consisted of 583 patients; 388 and 195 cases treated at SUS and HH respectively (Fig. 1). Clinical characteristics of the whole study cohort are shown in Table 1 comparing hospitals and Table 2 comparing antibiotic use.

**Table 1:** Clinical characteristics of the patient cohort divided into three groups total, patients treated at SUS and HH respectively

Variable	Total *n*=583	SUS *n*=388	HH *n*=195	P
Female n (%) Male n (%)	356 (61) 227 (39)	227 (56) 161 (44)	129 (66) 66 (44)	0.07
Age, years	61.0 (51-72)	61.0 (51-72)	61.0 (51-71)	0.94
Body Max Index	27.5 (24.7-30.5)	27.6 (25.8-30.5)	27.2 (24.2-30.4)	0.19
Previous diverticulitis n (%)	233 (40)	159 (41)	74 (38)	0.60
CT n (%)	506 (87)	320 (83)	186 (95)	<0.001
*CRP (mg/L)	87 (40-136)	84 (40-144)	90 (39-141)	0.54
Peak CRP (mg/L)	127 (80-183)	128 (77-186)	124 (86-172)	0.50
*WBC (x10^9^ cells/L)	12.4 (10.4-14.7)	12.5 (10.4-14.8)	12.3 (10.3-14.6)	0.16
Temperature (Celsius)	37.7 (37.2-38.2)	37.7 (37.2-38.2)	37.8 (37.3-38.3)	0.63
Antibiotics n (%)	437 (75)	345 (89)	101 (52)	<0.001
Recurrence n (%)	128 (22)	83 (21)	45 (23)	0.24
Complications n (%)	15 (3)	8 (2)	7 (4)	0.18
Charlson score	2 (1-4)	2 (1-4)	2 (1-4)	0.82
Hospital stay (days)	3 (2-4)	3 (2-4)	3 (2-4)	0.79
Colon evaluation within 6 months n(%)	430 (74)	280 (72)	150 (77)	0.22

*at admission, Values in median and IQR unless specified otherwise.

*CRP* C-reactive protein, *WBC* White Blood Cell count

**Table 2:** Clinical characteristics of the patient cohort divided into three groups total, and with and without antibiotics respectively

Variable	Total ***n***=583	Antibiotics ***n***=446	No antibiotics ***n***=137	P
**Female n (%)** **Male n (%)**	356 (61) 227 (39)	267 (60) 179 (40)	89 (65) 48 (35)	0.28
**Age, years median**	61 (51-72)	60 (50-71)	64 (54-73)	0.05
**Body Max Index**	27.8 (24.7-30.5)	27.2 (24.-30.9)	26.8 (24.3-29.8)	0.16
**Previous diverticulitis n**	233 (40)	181 (40.6)	52 (38.0)	0.58
**CT n (%)**	506 (87)	377 (85)	129 (94)	0.004
*CRP (mg/L)	87 (40-136)	90 (46-151)	65 (22-115)	0.016
**CRP (mg/L)**	127 (80-183)	138 (89-199)	97 (57-129)	< 0.001
*WBC (x10^**9**^ **cells/L)**	12.4 (10.4-14.7)	12.6 (10.6-15.1)	11.7 (9.9-13.9)	0.004
**Temperature (Celsius)**	37.7 (37.2-38.2)	37.8 (37.3-38.3)	37.6 (37.138.0)	0.011
**Recurrence n (%)**	127 (22)	96 (22)	31 (23)	0.87
**Complications n (%)**	15 (3)	11 (3)	4 (3)	0.77
**Charlson score**	2 (1-4)	2 (1-4)	2 (1-4)	0.07
**Hospital stay (days)**	3 (2-4)	3 (2-5)	3 (2-4)	<0.001
**Colon evaluation within 6 months n (%)**	430 (74)	338 (76)	92 (67)	0.04

*at admission, Values in median and IQR unless specified otherwise.

*CRP* C-reactive protein

*WBC* White Blood Cell count

Overall, 137 (24%) were managed without antibiotics and 437 (76%) with antibiotics. Forty-three patients (11%) at SUS and 94 patients (48%) at HH did not receive antibiotics during hospitalization (*p* < 0.001; Table 1). The AUD diagnosis was CT-verified in 320 patients (83%) at SUS compared to 186 patients (95%) at HH (*p* < 0.001). Colon evaluation follow-up was conducted in 430 (74%) of all cases with no statistically significant difference between SUS and HH (*n*=280, 72% vs *n*=150, 77%; *p* =0.22).

Patients who received antibiotics had higher median CRP levels both at admission (90 mg/L vs 65 mg/L; *p* = 0.016) and at peak (138 mg/L vs 97 mg/L; *p* < 0.001). The WBC was also higher (12.6 x109 cells/L vs 11.7 x109 cells/L; *p* = 0.004) in the antibiotics group. There was no significant difference in recurrences (22% vs 23%; *p* = 0.87), complications (3% in both groups; *p* = 0.77) or length of hospital stay, median 3 [2–4] days between the groups; *p*= 0.79.

During follow-up, two patients in the non-antibiotics group needed surgery compared to none in the antibiotic group (*p*=0.68). Of 506 patients with CT- verified AUD, 7 were diagnosed with colorectal cancer (1.4%) at follow-up. One of these was localized in the right colon, and one in transverse colon, one in rectum and the remaining in the sigmoid colon.

A multivariable regression analysis revealed that the variable (SUS or HH) used as a proxy for the use of protocol, or no protocol was strongly associated with the use of antibiotics [SUS OR 10.9 (CI 6.6-17.9) *p*=<0.001] Table 3.

**Table 3:** Multivariable regression analysis of factors related to non-antibiotic treatment in admitted acute uncomplicated diverticulitis patients

Variable	OR	CI	P
Age	1.1	0.7-1.9	0.60
CRP	1.0	1.0-1.0	<0.001
CT	0.5	0.2-1.1	0.09
Hospital	10.9	6.6-17.9	<0.001

*CRP* C-reactive protein

*CT* Computed Tomography

## Discussion

Non-antibiotic management for AUD was early described by Hjern et al. [13] and has thereafter been supported by two large RCTs [5, 6]. The present retrospective cohort study compared the adoption of this new knowledge to clinical practice at different hospitals. A significantly higher proportion of AUD patients were managed without antibiotics at the hospital with a standard treatment protocol (HH) compared to the hospital without a standard treatment protocol (SUS). Furthermore, the present study demonstrated that non-antibiotic management is safe for in majority of AUD patients. Complication rates were similar in both groups, and in accordance with other studies [4, 5].

To the best of our knowledge, the present study is unique in evaluating clinicians’ compliance to a new protocol of AUD management over time and comparing results to another hospital without a protocol. Unexpectedly physicians’ compliance at the reference hospital (HH) had declined compared to just after introduction of the protocol (60% vs 48% managed without antibiotics) [16]. However, at the hospital without a standard treatment protocol (SUS), only 11% of AUD patients were managed without antibiotics. The result reflects that the introduction and implementation of new knowledge in clinical practice is demanding even with the best effort. As shown by Cabana et al. [20] the lack of agreement and familiarity with guidelines, systemic tardiness including many different physicians being involved and unwillingness to change may be some reasons. Not surprisingly, the compliance to other presumed quality factors in AUD treatment such as CT-verification of the AUD diagnosis and follow-up colon evaluation were higher at HH compared to SUS. These results are likely to be generalizable to other Scandinavian settings.

Reasons for the low adherence to a treatment protocol or guidelines need further attention. Qualitative studies with interviews of clinicians may be one way forward.

Seven patients (1.4%) were diagnosed with a colorectal cancer within six months of discharge, all of which had a CT-verified AUD. A systematic review and meta-analysis, which investigated the role of routine colonic evaluation after radiologically confirmed acute diverticulitis, revealed that about 0.7% of patients with AUD, and 10.8 % of complicated diverticulitis had colorectal cancer [21]. Maintaining a regimen where AUD patients have a routine colonic follow up, may be necessary to detect misdiagnosis of colorectal cancer particularly in younger patients [22].

To date there is insufficient data regarding risk factors predicting a complicated course after an episode of AUD. Apart from high risk and non-admitted patients being excluded in this study, there was a selection bias, as patients with a high inflammatory response were more likely to receive antibiotics. In 2018, Bolkenstein et al. found that high CRP levels are a risk factor for failure of non-antibiotic management for AUD patients [23]. A recent study analysed the feasibility of non-antibiotic management in AUD reporting a failure rate of about 4% without identifying risk factors for failure [24]. Moreover, they concluded that most complications occurred in high-risk patients treated with antibiotics. Another meta-analysis has concluded that comorbidity is the only risk factor for treatment failure [25]. Consistently, many guidelines advise to avoid antibiotic therapy in immunocompetent, otherwise healthy AUD patients without systemic signs of infection [26, 27].

Historically AUD used to be diagnosed clinically but CT is now recommended both to diagnose AUD and to exclude complications [28, 29]. Although most of the AUD cases were CT-verified in both centres, there was a statistically significant difference (95% HH vs. 83% SUS). A high rate of CT imaging could be important in increasing the certainty of the diagnosis and thus reduce the use of antibiotic treatment.

A strength of this study is that it gives a true picture of clinical management of AUD in different hospitals, that are in the same region. Patients were followed consecutively, and the study is a follow-up of previously studied management of AUD patients. Limitations are mainly the retrospective study design. Patients were selected by diagnosis code and there is a risk for misdiagnosis at discharge and for patients being “missed” which should have been included. The ICD-codes could have been wider, including all diverticulitis codes to avoid this.

New evidence appears difficult to comply with and continuous reinforcement efforts are required to improve adherence. The study shows clearly that standard treatment protocols may help to implement new knowledge, but the treatment protocol alone is not enough to change a long-lasting treatment tradition. Future studies are needed to investigate the rationale and the reasons behind non-compliance to new treatment algorithms among physicians and concentrate on ways to facilitate implementation of new evidence in clinical practice. There are data advocating that training sessions, peer-review feedback programs, promoting comparative data sharing and engagement in the development of guidelines enhance compliance [30]. Applying a generic strategy to overcome barriers perceived by clinicians is a promising technique which has been shown to result in a 48% increase in adherence to new guidelines [31].

## Conclusion

The present study suggests that treatment protocols facilitate new treatment strategies and increase standard of care. Given the reported rates of adverse events following AUD, observational non-antibiotic management is considered safe in immunocompetent non-septic patients. The authors recommend the use of protocols to facilitate application. Furthermore, continuous efforts are required to assure adherence to new treatment protocols and reasons for low adherence warrants future studies. This is even more important now, as both the American Society of Colorectal Surgeons, and the European Society of Coloproctology have published new guidelines on diverticular disease within the last year, where the routine use of antibiotics is discouraged.

## Data Availability

The data that support the findings of this study are available from the corresponding author, NA, upon reasonable request.

## Abbreviations

RCT: Randomized control trial
CT: Computed tomography
AUD: Acute uncomplicated diverticulitis
HH: Helsingborg Hospital
SUS: Skåne University Hospital
